# Evidence of a Uniform Muscle-Tendon Unit Adaptation in Healthy Elite Track and Field Jumpers: A Cross Sectional Investigation

**DOI:** 10.3389/fphys.2019.00574

**Published:** 2019-05-15

**Authors:** Gaspar Epro, Steve Hunter, Matthias König, Falk Schade, Kiros Karamanidis

**Affiliations:** ^1^Sport and Exercise Science Research Centre, School of Applied Sciences, London South Bank University, London, United Kingdom; ^2^Olympic Training Center Rheinland, Cologne, Germany

**Keywords:** triceps surae, muscle strength, tendon stiffness, elite jumpers, athletic training

## Abstract

Different adaptive responses to mechanical loading between muscle and tendon can lead to non-uniform biomechanical properties within the muscle-tendon unit. The current study aimed to analyze the mechanical properties of the triceps surae muscle-tendon unit in healthy male and female elite track and field jumpers in order to detect possible inter-limb differences and intra-limb non-uniformities in muscle and tendon adaptation. The triceps surae muscle strength and tendon stiffness were analyzed in both limbs during maximal voluntary isometric plantar flexion contractions using synchronous dynamometry and ultrasonography in sixty-seven healthy young male (*n* = 35) and female (*n* = 32) elite international level track and field jumpers (high jump, long jump, triple jump, pole vault). Triceps surae muscle-tendon unit intra-limb uniformity was assessed using between limb symmetry indexes in the muscle strength and tendon stiffness. Independent from sex and jumping discipline the take-off leg showed a significantly higher (*p* < 0.05) triceps surae muscle strength and tendon stiffness, suggesting different habitual mechanical loading between legs. However, despite these inter-limb discrepancies no differences were detected in the symmetry indexes of muscle strength (5.9 ± 9.4%) and tendon stiffness (8.1 ± 11.5%). This was accompanied by a significant correlation between the symmetry indexes of muscle strength and tendon stiffness (*r* = 0.44; *p* < 0.01; *n* = 67). Thus, the current findings give evidence for a uniform muscle-tendon unit adaptation in healthy elite track and field jumpers, which can be reflected as a protective mechanism to maintain its integrity to meet the functional demand.

## Introduction

The ability to sprint and jump maximally requires high mechanical power outputs and energy generation capabilities at the joints of the lower extremity (Bobbert et al., 1986; Stefanyshyn and Nigg, 1998). Accordingly, the ability to maximize sport performance in jumping and sprint disciplines requires enhanced leg and ankle extensor muscle-tendon unit (MTU) capacities (e.g., muscular strength and tendon stiffness), where the muscle and tendon have to be considered as a functional unit to effectively generate high joint moment magnitudes and mechanical power output. Tendons can enhance muscle performance during stretch–shortening cycle activities (e.g., sprinting and jumping) due to their ability to reduce muscular work by storing and releasing elastic energy, which allows muscles to operate at more favorable velocities for force generation (Biewener and Roberts, 2000; Hof et al., 2002). Therefore, it is not surprising that the mechanical and morphological properties of both muscles and tendons have been shown to affect athletic performance (Kubo et al., 1999, 2011; Bojsen-Møller et al., 2005; Stafilidis and Arampatzis, 2007; Albracht and Arampatzis, 2013).

Previous longitudinal investigations demonstrate that increases in muscle strength are commonly accompanied by intermittent changes in tendon stiffness (Kubo et al., 2001, 2012; Arampatzis et al., 2007a,b, 2010; Kongsgaard et al., 2007; Bohm et al., 2014). These modifications in tendon stiffness may serve as a protective mechanism to maintain its integrity to meet the increased functional demand due to muscle strength changes. Nevertheless, recent observations demonstrate differences between muscle and tendon in the responsiveness to mechanical stimuli (Arampatzis et al., 2007a, 2010), time course of the adaptive mechanisms (Kubo et al., 2012) and the effectiveness of tissue turnover (Heinemeier et al., 2013). More specifically, muscles tend to have a superior plasticity in response to a wide range of exercise modalities (Moss et al., 1997; Schoenfeld et al., 2016) in comparison to tendons which seem to respond most effectively to a mechanical stimulus causing high tendon strain magnitudes over a longer time duration (Arampatzis et al., 2007a, 2010; Bohm et al., 2014). These adaptive differences within the MTU may be even more relevant for athletes experiencing high training loads in certain training modalities, especially in athletic disciplines with constant excessive plyometric loading, i.e., in volleyballers, basketballers and track and field jumpers. Plyometric training is, by its nature, a form of high magnitude mechanical loading (likely higher than in any resistance exercise), however, the time durations under which the forces cause tendon strain are rather short with ground contact times mostly under 200 ms during jumping. This could possibly explain why different plyometric loading regimens have not always demonstrated that rapid muscle strength adaptations (Saez de Villarreal et al., 2010) are accompanied by similar adaptive responses in tendons (Burgess et al., 2007; Kubo et al., 2007; Fouré et al., 2009, 2010; Houghton et al., 2013; Bohm et al., 2014; Hirayama et al., 2017). From a biomechanical perspective, an increase in muscle strength in the absence of compensatory-adaptive changes in tendon mechanical properties would place the tendon under a higher internal mechanical demand (i.e., higher strain), hence lead to a non-uniform adaptation within the MTU. Indeed, recent studies show that in adolescent athletes a two-fold stimulus of maturation in association with frequent and predominantly plyometric mechanical loading can lead to fluctuations in muscle strength without accompanying adaptive changes in the tendon (Mersmann et al., 2016). From a practical viewpoint this supports the suggestion that the total jumping volume is a risk factor for tendon overload injuries in elite athletes (Bahr and Bahr, 2014).

In particular, the Achilles tendon (AT) is predisposed for tendon injuries due to a considerably lower safety factor (ratio between ultimate failure stress and functional stress) in comparison to other tendons (Ker et al., 1988; Magnusson et al., 2001) and the prevalence of AT tendinopathies seems to increase after maturity in adult athletes (Cassel et al., 2018; Janssen et al., 2018). A recent study demonstrated higher m. triceps surae (TS) muscle strength and AT stiffness for the take-off leg in comparison to the swing leg in male collegiate track and field jumpers with somewhat higher inter-limb dissimilarities in AT stiffness in relation to muscle strength (Bayliss et al., 2016). Such asymmetrical adaptation could be the result of different habitual loading patterns between the limbs during plyometric exercises and may vary across different track and field jumping events due to differences in the event-specific demand (Deporte and Van Gheluwe, 1989; Perttunen et al., 2000; Plessa et al., 2010; Willwacher et al., 2017). Whether habitual mechanical loading, incorporating these potential discrepancies in various athletic disciplines would induce intra-limb non-uniformities between muscle and tendon adaptation remains unclear in elite jumping event athletes. Taking into account that the habitual mechanical loading may be higher in elite athletes, makes this suggestion plausible, however, there is a lack of sufficient evidence in the literature to suggest clear differences in intra-limb uniformity between elite athletes from different jumping disciplines. Moreover, it is not well-established whether such non-uniformities within the MTU due to habitual loading are potentiated in female elite athletes. Regarding this, it has been demonstrated that females, as well as exhibiting lower muscle strength and tendon stiffness, also show a diminished regulation of muscle and tendon remodeling in response to mechanical loading in comparison to male adults (Magnusson et al., 2007; Miller et al., 2007; Westh et al., 2008; Hansen and Kjær, 2014; McMahon et al., 2018).

Therefore, the aim of this cross-sectional investigation was to examine the inter-limb differences in TS muscle strength and AT stiffness and the intra-limb uniformity within TS MTU using symmetry indexes in healthy male and female elite track and field jumpers. It was hypothesized that in addition to potential leg-specific and sex-related differences in TS muscle strength and AT stiffness, both male and female elite jumping athletes would demonstrate intra-limb non-uniformities between muscle and tendon mechanical properties irrespective of jumping discipline.

## Materials and Methods

### Participants and Experimental Design

As a part of a nationwide study on the TS MTU adaptation, sixty-seven healthy young male (*n* = 35; age: 23 ± 4 years; body mass: 82 ± 7 kg; body height: 189 ± 7 cm; mean and standard deviation) and female (*n* = 32; age: 24 ± 4 years; body mass: 63 ± 6 kg; body height: 177 ± 7 cm) elite international level jumping event track and field athletes from the German national team voluntarily participated in the study. The athletes were divided into groups based on their specific athletic event (HJ, high jump; TJ, triple jump; LJ, long jump; PV, pole vault; Supplementary Table 1). Exclusion criteria included any previous AT ruptures and any tendon problems (tendinopathy, etc.) within the last 6 months, which could have potentially influenced the findings. The study was approved by the ethics committee of the German Sport University Cologne and prior to commencing the study all participants gave written informed consent in accordance with the Declaration of Helsinki.

In all athletes the TS MTU mechanical properties (maximal ankle plantar flexion moment and AT stiffness) were assessed in both legs, as a part of a daily training session, at their respective Olympic training centers or at the National Team training camps during or directly prior to the competition period. The preferred jumping leg was defined as the take-off leg, whereas the contralateral non-jumping leg was defined as the swing leg. For triple jumpers the hop leg was considered as the take-off leg. TS MTU intra-limb uniformity was assessed using between limb symmetry indexes of maximal ankle plantar flexion moment and AT stiffness.

### Analysis of Triceps Surae Muscle Strength and Achilles Tendon Stiffness

The maximal ankle plantar flexion moment and AT stiffness were assessed in all participants using synchronous ultrasonography and dynamometry on a custom-made device. The analysis methods for TS MTU properties have been described in more detail in a previous study (Ackermans et al., 2016). Briefly, the participants were seated with their ankle and knee joints fixed at 90° angle (thigh and foot perpendicular to the shank) and their foot on a custom-made strain gauge type dynamometer (1000 Hz; TEMULAB^®^, Protendon GmbH & Co. KG, Aachen, Germany). Each participant’s foot was positioned by setting the midpoint of the malleolus lateralis in line with the dynamometer’s axis of rotation using the aid of a laser-guided electrical potentiometer system (Ackermans et al., 2016). Prior to the measurements, and in order to “precondition” the tendon, each athlete performed their individualized warm-up routine, followed by a standardized warm-up program of 2–3 min of submaximal and three maximal isometric contractions (Maganaris, 2003). All warm-up contractions were guided by the TEMULAB software.

In order to examine the maximal ankle plantar flexion moment and the force–elongation relationship of the tendon during the loading phase, all athletes performed isometric plantar flexion contractions at different force levels consisting of: three maximal voluntary ankle plantar flexion contractions (MVC) with verbal encouragement, followed by three sustained contractions at 30, 50, and 80% of the maximal joint moment determined during the MVC measurements. The individual maximal ankle plantarflexor muscle strength (TS muscle strength) was normalized to the athlete’s body mass (Nm/kg) to ensure an appropriate comparison between subject groups. During all sustained contractions the participants were guided by a visual feedback of the produced joint moment on a computer screen. The resultant joint moments acting about the ankle joint were calculated using inverse dynamics, accounting for the gravitational moments using a prior passive measurement (relaxed muscle in the fixed position). By aligning the axis of rotation of the ankle with the dynamometer’s axis of rotation, the ankle joint moment could be considered equal to the moment of the force plate (Ackermans et al., 2016). It is important to note, that the resultant ankle plantar flexion moment is an approximation of the moment produced by the TS muscle, because it does not account for the individual moment contributions of the other synergistic agonist muscles or the antagonist dorsiflexors. The AT force was calculated by dividing the resultant ankle joint moment by the tendon moment arm obtained from the literature (Maganaris et al., 1998).

Following the preconditioning of the tendon, a laser-guided electrical potentiometer system recording linear displacement was used to determine the AT resting length as the distance between the most proximal point of the tuber calcanei and the myotendinous junction (MTJ) of the m. gastrocnemius medialis (both defined using ultrasonography). The displacement of the MTJ of the m. gastrocnemius medialis during the contractions was analyzed using a securely positioned 40 mm linear array ultrasound probe (27 Hz; MyLab^TM^One, Esaote; Genoa, Italy) and by manually digitizing MTJ in the TEMULAB software (Protendon GmbH & Co. KG, Aachen, Germany). A casing with adjustable straps was used to fix the ultrasound probe on the shank above the MTJ to prevent any movement in relation to the skin. The MTJ displacement was digitized at rest (0% MVC) and at the three sustained contractions (target joint moment held by the participant for 3 s) at the predetermined target ankle joint moment levels (30, 50, and 80% MVC). A specific trial was repeated, when the athlete failed to hold a steady state of 3 s at a range of ±5% of the target joint moment. The tendon elongation at maximal ankle joint moment (100%) was calculated via a linear extrapolation of the elongation at 50 and 80% target joint moments (Ackermans et al., 2016), because instructions (given loading rate and holding the force a certain level) during the maximal plantar flexion contraction may restrict participants ability to contract maximally. This extrapolated approach may play merely a small role for the determined maximal tendon strain as more than half of the entire elongation is achieved within the first 25% of the MVC and consequently only quite small tendon length changes occur between 80 and 100% of the MVC. The reason for using sustained contractions in determining the tendon elongation was to account for potential effects of the tendon viscoelasticity (loading rate dependency) on the force-elongation relationship of the human AT in vivo. For example if a set time (e.g., 2 s) is given to reach maximum force, the absolute loading rate will differ between participants or legs of different strengths. The theoretical consideration of the current approach is that the sustained method may negate loading rate dependency as it accounts for the phase shift due to the time-dependent viscous properties of the viscoelastic material (Meyers and Chawla, 1999). Regarding this issue, in a recent study (McCrum et al., 2018) loading rate effects up to 25% of the MVC were seen during plantar flexion contractions, which were reduced by the sustained method. The day-to-day reliability of this method to assess AT stiffness has been proven previously (Ackermans et al., 2016), by showing no significant differences between trials and with the mean of the individual ratios of tendon stiffness between days laying close to 1. To account for the effect of an inevitable ankle joint angular rotation on the measured tendon elongation during each contraction (Muramatsu et al., 2001), the changes in ankle joint angle were calculated via the inverse tangent of the ratio of the heel lift (measured with a potentiometer) to the distance between the ankle joint axis and the head of the fifth metatarsal bone (Ackermans et al., 2016). The AT stiffness was calculated as the ratio between the change in the calculated tendon force and change in the resultant tendon elongation between 30 and 80% of maximum tendon force. For more detailed information, including potential methodological drawbacks of the TS MTU analysis and the validity of using sustained contractions for assessing tendon stiffness, please see supplement material in Ackermans et al. (2016) and McCrum et al. (2018).

### Analysis of Inter-Limb Symmetry Within the TS MTU

As mentioned above, improvements in muscle strength (increased functional demand on the tendon) are commonly accompanied by intermittent changes in tendon stiffness. In order to examine whether a long-term habitual athletic training leads to a uniform adaptation in the TS muscle strength and AT stiffness between the take-off and swing leg, the symmetry index (SI) was determined (Robinson et al., 1987) between the limbs as follows:

(1) $$SI=\frac{X_{TakeoffLeg}-X_{SwingLeg}}{\frac{1}{2}\left(X_{TakeoffLeg}+X_{SwingLeg}\right)}\times 100\%$$

where X_TakeoffLeg_ is the parameter from the take-off leg and X_SwingLeg_ the corresponding parameter from the swing leg. Accordingly, a positive symmetry index means that the selected parameter has a greater value in take-off leg than swing leg, and a negative symmetry index means that the value is higher in swing leg. Similar SI of TS muscle strength and AT stiffness would illustrate a uniform adaptation within TS MTU (intra-limb uniformity), whereas differences in SIs would indicate to a non-uniform adaptation between muscle and tendon in either take-off or swing leg.

### Statistics

The Shapiro-Wilk and Levene’s test was used to confirm the normality of distribution and homogeneity of variance of the data (*p* > 0.05). A three-way repeated measures analysis of variance (ANOVA) with leg (take-off vs. swing leg) and jumping discipline (high jump, triple jump, long jump, pole vault) and sex (male vs. female) as factors was performed to examine potential differences between subject groups and legs in TS muscle strength, AT stiffness and maximal AT strain. Potential differences between the SIs were analyzed using an additional three-way ANOVA [sex and discipline as factors and SI of TS muscle strength (SI_Moment_) and SI of AT stiffness (SI_Stiffness_) as parameters]. In order to identify possible differences in age, body mass, and body height a two-way ANOVA was implemented with sex and jumping discipline as factors. In the case of a detected significant main effect or interaction a Bonferroni *post hoc* comparison was performed. A Pearson product-moment correlation coefficient was used to examine the relationship between TS muscle strength and AT stiffness in the take-off and swing leg separately for male and female jumpers as well as the relationship between the symmetry indexes (SI_Moment_ and SI_Stiffness_) using all analyzed elite jumpers. The level of significance was set at α = 0.05 and all statistical analyses were performed using Statistica (Release 10.0; StatSoft Inc., Tulsa, OK, United States). All results in the text and figures are provided as means and standard deviation (SD).

## Results

### TS MTU Mechanical Properties in Elite Track Field Jumpers

**FIGURE 1:**
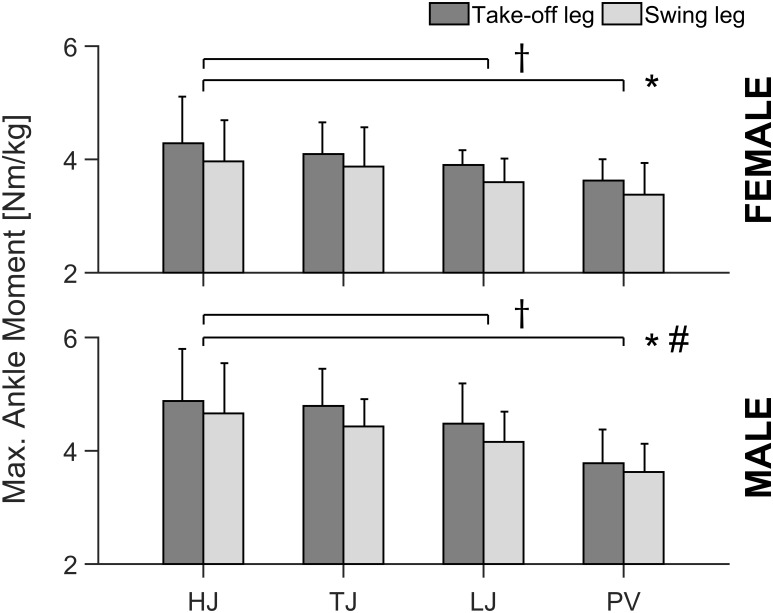
Maximal ankle plantar flexion moment (Max. Ankle Moment) of the take-off and swing leg in all analyzed specific jumping event groups (HJ, high jump; TJ, triple jump; LJ, long jump; PV, pole vault) for both male and female elite athletes. All values are expressed as means with SD (error bars). ^∗^Statistically significant difference between take-off and swing leg (*p* < 0.05). ^#^Statistically significant difference between male and female elite athletes (*p* < 0.05). ^†^Statistically significant difference to PV (*p* < 0.05).

The implemented three-way repeated measures ANOVA showed a significant leg effect (*p* < 0.001) in TS muscle strength with higher values for the take-off in comparison to swing leg independent of jumping discipline (Figure 1; no significant interaction). There was a jumping discipline effect (*p* = 0.006), with pole vaulters (PV) showing significantly lower TS muscle strength in comparison to high (HJ; *p* = 0.001), long (LJ; *p* = 0.009) and triple jumpers (TJ; *p* = 0.028) irrespective of athletes’ sex and analyzed leg (Figure 1). Furthermore, male jumpers showed significantly (*p* = 0.002) higher TS muscle strength compared to female jumpers (Figure 1).

**FIGURE 2:**
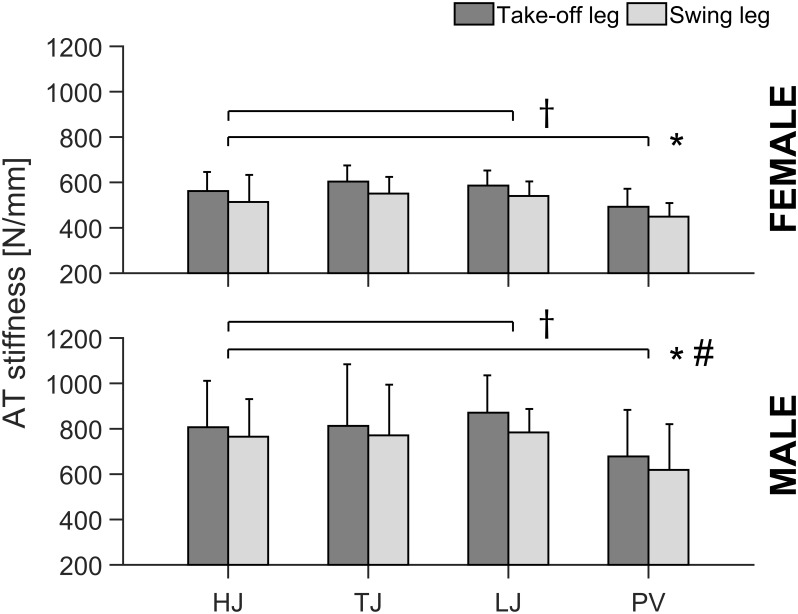
Achilles tendon (AT) stiffness of the take-off and swing leg in all analyzed specific jumping event groups (HJ, high jump; TJ, triple jump; LJ, long jump; PV, pole vault) for both male and female elite athletes. All values are expressed as means with SD (error bars). ^∗^Statistically significant difference between take-off and swing leg (*p* < 0.05). ^#^Statistically significant difference between male and female elite athletes (*p* < 0.05). ^†^Statistically significant difference to PV (*p* < 0.05).

**FIGURE 3:**
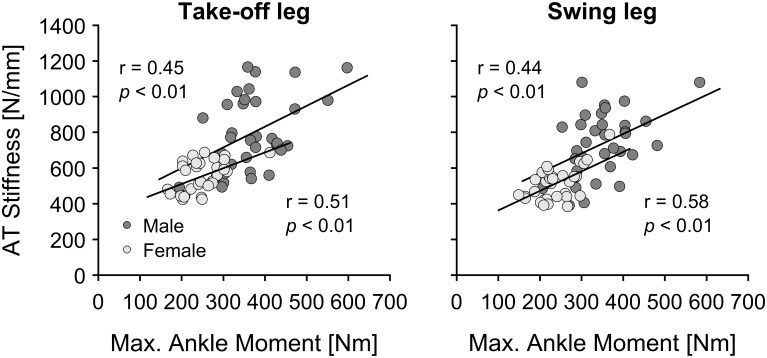
Correlations between maximal ankle plantar flexion moment (Max. Ankle Moment) and Achilles tendon (AT) stiffness in the take-off and swing leg in male (*n* = 35) and female (*n* = 32) elite athletes. Correlation coefficients for male elite jumpers (dark gray circles) are shown in the upper left corner and for female elite jumpers (light gray circles) in the lower right corner of the figure.

For the AT stiffness, the ANOVA revealed a significant leg effect (*p* < 0.001) with greater AT stiffness for the take-off leg in comparison to swing leg, irrespective of the jumping discipline and sex (Figure 2). Similar to TS muscle strength, there was a jumping discipline effect (*p* = 0.037) in AT stiffness with lower values for the PV in comparison to all other jumping disciplines (*p* = 0.038, *p* = 0.035, and *p* = 0.041, respectively, for HJ, LJ, and TJ), irrespective of athletes’ sex and analyzed leg (Figure 2). Moreover, male in comparison to female jumpers showed significantly (*p* < 0.001) higher AT stiffness (Figure 2). There were no significant main effects or interactions in maximal AT strain (average values across all disciplines and sex: take-off leg 4.6 ± 1.0 vs. swing leg 4.8 ± 1.1%). Statistically significant correlations (*p* < 0.01) were detected between TS muscle strength and AT stiffness in both male (*n* = 35; 0.44 ≤ *r* ≤ 0.45) and female (*n* = 32; 0.51 ≤ *r* ≤ 0.58) jumpers irrespective of the analyzed leg (Figure 3).

### Inter-Limb Symmetry in TS MTU Mechanical Properties in Elite Track Field Jumpers

**FIGURE 4:**
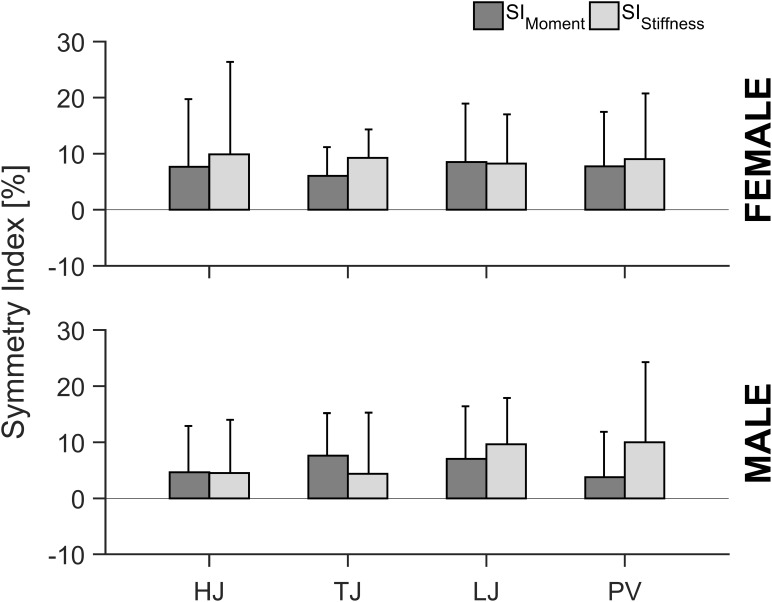
Symmetry index of the maximal ankle plantar flexion moment (SI_Moment_) and Achilles tendon stiffness (SI_Stiffness_) in all analyzed specific jumping event groups (HJ, high jump; TJ, triple jump; LJ, long jump; PV, pole vault) for both male and female elite athletes. All values are expressed as means with SD (error bars). A positive symmetry index displays a greater value for the take-off leg and a negative symmetry index for the swing leg.

**FIGURE 5:**
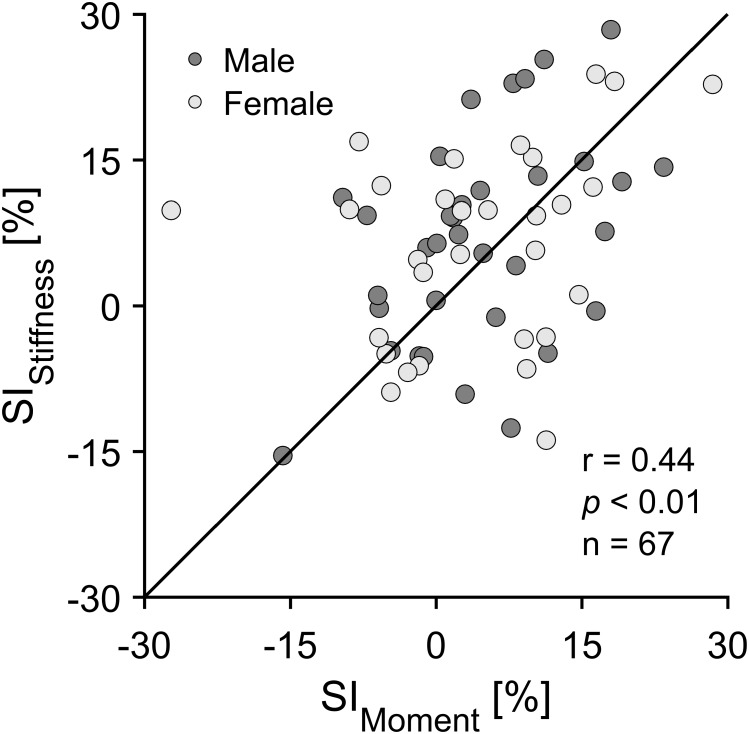
Correlation between the symmetry index of the maximal ankle plantar flexion moment (SI_Moment_) and Achilles tendon stiffness (SI_Stiffness_) in all analyzed elite athletes. Correlation coefficients were comparable for male elite jumpers (dark gray circles; *r* = 0.44; *p* < 0.01; *n* = 35) and female jumpers (light gray circles; *r* = 0.44; *p* = 0.01; *n* = 32). A positive symmetry index displays a greater value for the take-off leg and a negative symmetry index for the swing leg.

The implemented ANOVA revealed no significant differences between SI_Moment_ and SI_Stiffness_ independent of sex and specific athletic discipline (Figure 4). The SI of TS muscle strength (SI_Moment_) values over all the analyzed elite jumpers ranged from -15.8 to 28.5% with an average value of 5.9 ± 9.4% (positive values indicate take-off leg dominance; Figure 4). For the SI of AT stiffness (SI_Stiffness_) values in elite jumpers ranged from -15.4 to 36.6% with an average value of 8.1 ± 11.5% (positive values indicate higher stiffness for the take-off leg; Figure 4). Statistically significant correlations (*r* = 0.44; *p* < 0.01; *n* = 67) were identified between SI_Moment_ and SI_Stiffness_ in both male and female jumpers (Figure 5).

## Discussion

This study investigated TS MTU mechanical properties in both legs of healthy male and female elite track and field jumpers to detect possible inter-limb differences and intra-limb non-uniformities in TS muscle strength and AT stiffness due to habitual athletic training. It was hypothesized that elite jumpers will display possible intra-limb non-uniformities between TS muscle strength and tendon stiffness along with potential leg-specific differences in muscle and tendon mechanical properties. However, the hypothesis could not be confirmed, as the leg-specific differences were not accompanied by intra-limb non-uniformities within the MTU, irrespective of jumping event and sex.

Despite resistance exercise interventions inducing high tendon strains frequently reporting concurrent changes in tendon biomechanical properties along with increased muscle strength (Kubo et al., 2001; Arampatzis et al., 2007a; Kongsgaard et al., 2007), purely plyometric exercise interventions show inconsistent results for tendon adaptation in comparison to skeletal muscle (Burgess et al., 2007; Kubo et al., 2007; Fouré et al., 2009, 2010; de Villarreal et al., 2010; Houghton et al., 2013; Bohm et al., 2014; Hirayama et al., 2017). A reasonable explanation for this could be that tendons may not respond as effectively to loading profiles characterized by short tendon strain durations (Bohm et al., 2014), i.e., plyometric exercises such as repetitive jumping. Nevertheless, the findings of the current study detected a greater AT stiffness (on average ∼9%) along with higher TS muscle strength (∼7%) for the take-off leg in comparison to the swing leg in elite track and field jumpers. Accordingly, the current cross-sectional investigation does not give evidence to suggest that habitual loading in elite jumpers leads to a clear non-uniform adaptation within the MTU as previously shown in adolescent athletes (Mersmann et al., 2016). This is further reflected by the significant correlation between TS muscle strength and tendon stiffness for both male and female elite jumpers (Figure 3). Our identified leg-specific differences may relate to a possibly greater total unilateral jumping volume and hence an overall increased mechanical loading of the take-off leg during the training and competition. Similar asymmetric adaptations in muscle and tendon mechanical properties due to habitual loading have been identified also in other sport disciplines (e.g., badminton, fencing, handball) (Couppé et al., 2008; Hansen et al., 2013) and are in line with observations from collegiate-level jumping athletes (Bayliss et al., 2016).

Such a concurrent between limb increase in tendon stiffness and muscle strength in elite athletes may be considered as a protective mechanism to sustain tendon integrity in order to withstand the functional demand from the muscle (Kubo et al., 2001; Arampatzis et al., 2007a; Kongsgaard et al., 2007). Moreover, when considering the relative differences in MTU mechanical properties, we found comparable SIs in muscle strength and tendon stiffness. These findings were irrespective of the athletic discipline or sex (Figure 4), indicating that the overall cumulative habitual loading in elite jumping event athletes does not lead to intra-limb non-uniform adaptation within the TS MTU. This suggestion is further supported by the similar maximal AT strain values between take-off and swing leg and the significant correlations between SI_Moment_ and SI_Stiffness_ (Figure 5). Moreover, the above findings were independent of sex, indicating the possible different regulation of muscle and tendon remodeling in response to mechanical loading (Magnusson et al., 2007; Miller et al., 2007; Westh et al., 2008; Hansen and Kjær, 2014; McMahon et al., 2018) seems not to affect the uniform development of the TS MTU in female elite athletes. However, one might suggest that our reported inter-limb differences in elite athletes might be a result of biological variation and not due to habitual mechanical loading. Regarding this, it is important to note that in an additional study we analyzed a group of elite international and national level male sprinters (100 m personal best in a range from 10.15 to 10.89 s; mean 10.64 ± 0.23 s) and detected clearly lower intra-limb non-uniformities (on average 0.1 ± 12.3 and 3.3 ± 8.9%, respectively, for the SI_Moment_ and SI_Stiffness_, with take-off leg defined as the front limb in the block start). Based on the current findings we conclude that elite track and field jumpers experienced different habitual mechanical loading profiles between limbs during training and competitions that leads to higher TS muscle strength and AT stiffness for the take-off leg in comparison to the swing leg. However, the magnitude of differences seems not to cause any measurable non-uniform adaptation within the TS MTU regardless of athletic discipline and sex.

From a biomechanical perspective, when increases in muscle strength are not accompanied by increases in tendon stiffness, an imbalance within the muscle-tendon unit may develop, described by a raise in the mechanical demand for the tendon, possibly making it more susceptible for injuries. Opposite to variations in ultimate stress (i.e., stress at tendon failure) between different tendons, ultimate tendon strain is shown to be rather constant (LaCroix et al., 2013). Accordingly, based on previous experimental data from cadaver studies and animal models (Wren et al., 2003; LaCroix et al., 2013), habitual tendon strain can be considered as a central indicator for tendon mechanical demand and the risk of injury. Thus, a uniform adaptation in both muscular strength as well as in tendon stiffness needs to be assured to maintain tendon homeostasis, which may be of particular relevance for the AT due to its low safety factor. Although in the current study we were not able to identify any non-uniformities within the TS MTU, it is important to mention that the measurements of the examined elite track and field athletes were taken in a specific time period (during or directly prior of the competition period). Therefore, we cannot answer whether there are potential fluctuations in maximal ankle plantar flexion moment and AT stiffness throughout the athletic training over time in elite athletes that would affect our inter- and intra-limb comparison. In relation to this, it is important to note that both male and female jumpers showed significant, albeit moderate correlation coefficients between TS muscle strength and AT stiffness as well as SI_Moment_ vs. SI_Stiffness_ (0.44 ≤ *r* ≤ 0.58; Figure 3, 5). These findings may indicate that intra- and inter-limb fluctuations in muscle strength during athletic training may not always be accompanied by similar adaptive changes in tendon stiffness, hence potential discordances in muscle and tendon adaptation cannot be excluded in some elite athletes. The above suggestion is supported by the observation that the current pool of elite athletes showed relatively high inter-subject variations in the symmetry indexes and are in line with earlier reports of large individual leg-differences in muscle architecture of m. gastrocnemius medialis in national level jumpers (Aeles et al., 2017). Accordingly, based on the above provided biomechanical perspective for an increased risk of tendon injuries, future studies should investigate longitudinal adaptations of the TS MTU mechanical properties in order to examine whether elite jumpers show similar fluctuations in muscle strength capacities without parallel or compensating changes in tendon stiffness as previously reported in adolescence athletes (Mersmann et al., 2016). This would provide an important information for athletes-coaches and their support teams about the continuous adaptation processes during various phases of athletic training and whether the uniformity within MTU is disrupted.

One might argue that the knee joint being flexed at 90° could place the gastrocnemius muscle in a less favorable position to generate force, which may result in differences of tendon deformation between its subparts, hence influencing the calculated tendon stiffness. The main reasoning to use this joint angle setup was to reduce the inevitable ankle joint angular rotation in comparison to fully-extended knee joint angle (Ackermans et al., 2016) as it leads to a considerable overestimation of the tendon elongation due to the exerted force during the plantar flexion contraction. In a previous study (Ackermans et al., 2016) we used in addition a more dorsiflexed ankle joint position (85° joint angle) with the same knee joint configuration to improve the force potential of the gastrocnemii and their contribution to the net joint moment. Although, we found significantly higher ankle joint moments and tendon elongation for the more dorsiflexed position, there were no significant differences in tendon stiffness between the two setups (Ackermans et al., 2016 Supplemental Data). Therefore, even though a flexed knee joint may change the complex interactions between different subparts of the AT, it seems not to lead to changes in the force-length relationship of the m. gastrocnemius medialis tendon during loading, especially in its “linear part” where the tendon stiffness was calculated. Finally, moment arms from the previous literature were used to calculate the AT force (Maganaris et al., 1998) instead of assessing individual moment arms, which could influence the current AT stiffness values in absolute terms. However, while this may affect the comparison between male and female athletes we believe that this drawback may not significantly affect the main outcomes measures of the current study in terms of inter-limb symmetries and intra-limb uniformity as AT moment arms have been previously shown to be symmetrical between limbs (Bohm et al., 2015).

## Conclusion

In conclusion, the current findings demonstrate a higher TS muscle strength and greater AT stiffness in the take-off in comparison to swing leg in healthy male and female elite track and field jumpers. Moreover, these inter-limb differences were independent of jumping discipline and athlete sex, suggesting that the limbs of elite jumpers experience different habitual mechanical loading profiles during training and competition. However, the magnitude of differences seems not to cause any measurable non-uniform adaptation within the TS MTU, which can be reflected as a protective mechanism to maintain its integrity to meet the functional demand.

## Ethics Statement

The study was approved by the ethics committee of the German Sport University Cologne and prior to commencing the study all participants gave written informed consent in accordance with the Declaration of Helsinki.

## Author Contributions

GE, FS, and KK participated in the conception and design of the research. GE, FS, and MK performed the experiments. GE, SH, and KK analyzed the data. GE, SH, MK, FS, and KK interpreted results of experiments. GE and KK prepared the figures and drafted manuscript. GE, SH, MK, FS, and KK edited and revised the manuscript and approved final version of manuscript.

## Conflict of Interest Statement

KK has equity in Protendon GmbH & Co. KG, whose measurement device and software was used for the data processing and analysis in this study. The remaining authors declare that the research was conducted in the absence of any commercial or financial relationships that could be construed as a potential conflict of interest.
