# Supplementary material for: Evidence of a Uniform Muscle-Tendon Unit Adaptation in Healthy Elite Track and Field Jumpers: A Cross Sectional Investigation
Source: Front Physiol. 2019 May 15;10:574. doi: 10.3389/fphys.2019.00574 (PMC6529647; doi:10.3389/fphys.2019.00574)
Supplement: Supplementary file 1 [file Table_1.DOCX]

Supplementary Material

Evidence of a uniform muscle-tendon unit adaptation in healthy elite track and field jumpers: a cross sectional investigation

Gaspar Epro^1^, Steve Hunter^1^, Matthias König^1^, Falk Schade^2^, Kiros Karamanidis^1*^

^1^Sport and Exercise Science Research Centre, School of Applied Sciences, London South Bank University, London, United Kingdom

^2^Olympic Training Center Rheinland, Cologne, Germany

*** Correspondence:**Kiros Karamanidis
k.karamanidis@lsbu.ac.uk

**Table 1**: Anthropometrics and personal best results of the participating male and female elite jumpers

|  | **High jump** | **Triple jump** | **Long jump** | **Pole vault** |
| --- | --- | --- | --- | --- |
| Subjects (n) | 11 M  11 F | 6 M  5 F | 9 M  8 F | 9 M  8 F |
| Age (years) | 24 ± 5  23 ± 3 | 22 ± 3  23 ± 4 | 22 ± 3  24 ± 4 | 22 ± 4  25 ± 6 |
| Body height (cm) | 195 ± 4  182 ± 6 | 187 ± 8  177 ± 5 | 185 ± 6  177 ± 5 | 187 ± 4  172 ± 8 |
| Body mass (kg) | 82 ± 7  65 ± 5 | 87 ± 7  60 ± 5 | 80 ± 9  64 ± 7 | 81 ± 6  62 ± 7 |
| BMI (kg/m^2^) | 21.6 ± 1.8  19.7 ± 1.1 | 24.8 ± 3.6  19.2 ± 0.6 | 23.2 ± 2.0  20.4 ± 1.7 | 23.2 ± 1.3  21.0 ± 1.4 |
| Personal best result (m) | 2.27 ± 0.05  1.90 ± 0.05 | 16.43 ± 0.60  13.81 ± 0.76 | 8.01 ± 0.26  6.72 ± 0.20 | 5.66 ± 0.21  4.58 ± 0.22 |

Values are expressed as means ± SD. M, male; F, female; BMI, body mass index; TS, triceps surae; AT, Achilles tendon.
